# Networks that link cytoskeletal regulators and diaphragm proteins underpin filtration function in *Drosophila* nephrocytes

**DOI:** 10.1016/j.yexcr.2018.02.015

**Published:** 2018-03-15

**Authors:** Simi Muraleedharan, Aksah Sam, Helen Skaer, Maneesha S. Inamdar

**Affiliations:** a Molecular Biology and Genetics Unit, Jawaharlal Nehru Centre for Advanced Scientific Research, Jakkur, Bangalore 560064, India; b Department of Zoology, University of Cambridge, Downing Street, Cambridge CB2 3EJ, UK; c Institute for Stem Cell Biology and Regenerative Medicine, GKVK, Bellary Road, Bangalore 560065, India

**Keywords:** Nephrocyte, Podocyte, Cytoskeleton, Slit diaphragm, Kidney function

## Abstract

Insect nephrocytes provide a valuable model for kidney disease, as they are structurally and functionally homologous to mammalian kidney podocytes. They possess an exceptional macromolecular assembly, the nephrocyte diaphragm (ND), which serves as a filtration barrier and helps maintain tissue homeostasis by filtering out wastes and toxic products. However, the elements that maintain nephrocyte architecture and the ND are not understood. We show that *Drosophila* nephrocytes have a unique cytoplasmic cluster of F-actin, which is maintained by the microtubule cytoskeleton and Rho-GTPases. A balance of Rac1 and Cdc42 activity as well as proper microtubule organization and endoplasmic reticulum structure, are required to position the actin cluster. Further, ND proteins Sns and Duf also localize to this cluster and regulate organization of the actin and microtubule cytoskeleton. Perturbation of any of these inter-dependent components impairs nephrocyte ultrafiltration. Thus cytoskeletal components, Rho-GTPases and ND proteins work in concert to maintain the specialized nephrocyte architecture and function.

## Highlights

• *Drosophila* nephrocytes have a unique cytoplasmic cluster of F-actin.
• Microtubules, Rho-GTPases and endoplasmic reticulum position the actin cluster.
• Nephrocyte diaphragm proteins localize to and regulate actin cluster organization.
• Perturbation of any of these inter-dependent components impairs ultrafiltration.

## 1 Introduction

Chronic kidney disease resulting from gradual loss of ultrafiltration function due to genetic predisposition or disorders such as diabetes and hypertension is a major healthcare problem. The kidney podocyte has a complex morphology that is essential for its role as a glomerular filter. Slit diaphragm (SD) proteins and extracellular matrix-binding transmembrane receptors that make up the filtration diaphragm are coupled to the actin cytoskeleton via integral membrane proteins [1], [2], [3], [4], [5]. The actin cytoskeleton is a key component that regulates podocyte shape and function. While microtubules (MTs) and intermediate filaments (IFs) provide the major structural support in the podocyte cell body and primary processes [6], [7], dense arrays of actin microfilaments are present in the foot processes [8]. In several nephrotic syndromes this differential distribution of cytoskeletal components is lost, leading to loss of SD integrity, foot process effacement and proteinuria [9], [10], [11], [12], [13], [14], [15], [16], [17]. RhoGTPase activity orchestrates actin organization and thereby podocyte function [18], [19], [20]. However, the role of MTs and IFs in regulating the actin cytoskeleton for podocyte function is not well studied.

Recent studies have shown that the *Drosophila* nephrocyte is functionally homologous to the kidney podocyte, providing an excellent molecular-genetic model for elucidating mechanisms that regulate podocyte ultrastructure and function [21], [22], [23], [24]. *Drosophila* nephrocytes consist of two groups, namely pericardial cells (PCs) flanking the cardiac tube and garland cells (GCs), which lie above the proventriculus [25], [26]. Nephrocytes carry out ultrafiltration and sequestration of macromolecules, metabolic wastes and toxins from the hemolymph [26], [27], [28]. Functional assays of ultrafiltration and lifespan in various *Drosophila* mutants showed that nephrocytes have a filtration diaphragm (nephrocyte diaphragm, ND) that functions in a charge and size selective manner similar to the podocyte SD. The ND is composed of proteins homologous to those that construct the podocyte SD: Dumbfounded (Duf; Neph1 homolog) and Sticks and Stones (Sns; Nephrin homolog) [22]. Phosphorylated Duf interacts with *Drosophila* Nck (Dreadlocks) for downstream signaling [21].

The nephrocyte plasma membrane forms extensive invaginations, which resemble the foot processes of podocytes [25]. However, the organization and contribution of various cytoskeletal components in maintaining nephrocyte architecture and function is not known. Understanding these details will make the *Drosophila* nephrocyte a more powerful and valuable model for studying podocyte dysfunction and kidney diseases. Towards this aim, we initiated analysis of factors that regulate nephrocyte architecture and function. Here, we show that nephrocyte actin organizes into a unique structure whose position is regulated by the MTs, endoplasmic reticulum (ER), Rho-GTPases (Rac1 and Cdc42) and the ND proteins, Sns and Duf. In addition, we show that, while MTs and Sns mutually regulate each other's expression and localization, Duf does not play a major role in maintaining the MT cytoskeleton. Taken together, our results demonstrate reciprocal regulation between cytoskeletal components and ND proteins that is essential for size and charge-dependent ultrafiltration.

## 2 Results

### 2.1 Nephrocyte actin is located both cortically and as a central cluster

**Fig. 1:**
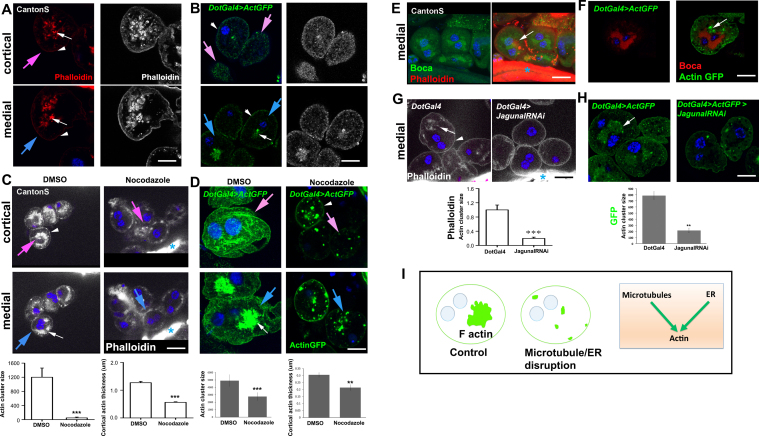
Nephrocytes have cortical actin and a unique actin cluster organization maintained by microtubules and ER. A representative cell at the cortical (pink arrow) or medial (blue arrow) Z slice is indicated. White arrows: actin cluster; arrowheads: cortical actin. (A-B) Whether marked by Phalloidin [in Canton S (A, C, E) or in *DotGAL4* (G)] or by the expression of ActinGFP (green in *DotGal4*>*UASActinGFP*) (B, D, F, H), Actin is seen at the cell cortex (arrowhead) and as a cytoplasmic cluster (white arrows) (Grey scale images in A, B represent the fine edges of actin structures adjusted using ImageJ software). (C, D) Endogenous Actin (Phalloidin) (C) or ActinGFP (green) (D) organization after treatment with DMSO and Nocodazole. Graph shows quantification of actin cluster size and cortical actin thickness (see Materials and Methods). (E, F) Endogenous Actin (Phalloidin) (E) or ActinGFP (green) (F) and ER lumen marker, Boca co-staining. Actin cluster lies in close proximity to the ER. (G, H) Endogenous Actin (Phalloidin) (G) or ActinGFP (green) (H) organization in control and Jagunal RNAi nephrocytes. Blue asterisks indicate the proventriculus. Graph shows quantification of actin cluster size. (I) Model shows disintegration of actin cluster in cells with MTs or ER disrupted. n ≥ 30 cells from three independent experiments. Error bars depict ± SEM. **p < 0.01, ***p < 0.001 Scale bars: (A, B, D, F, H) 10 µm (C, E, G) 20 µm.

**Fig. S1:**
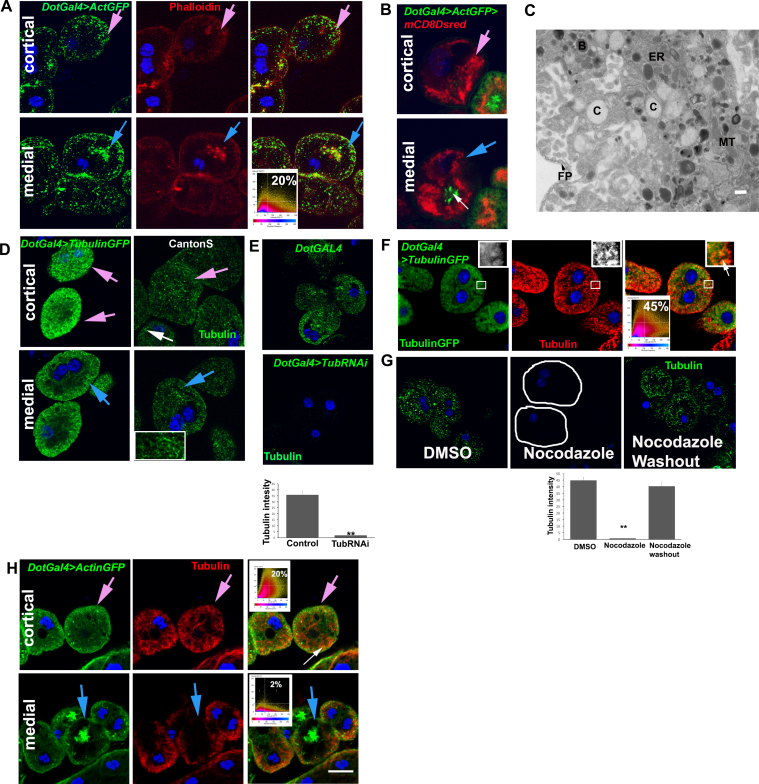
Analysis of nephrocyte actin upon disrupting microtubules and ER. A representative cell at the cortical (pink arrow) or medial (blue arrow) Z slice is indicated. (A) Co-localization of Actin GFP (green) and Phalloidin (red) in nephrocytes. Inset showing the co-localization index. (B) Membrane (red) and actin (green) in nephrocytes expressing *DotGAL4>ActinGFP>mCD8 dsred* showing that actin cluster is not localized to the cell membrane. (C) Electron micrograph of a wild type nephrocyte showing the subcellular organization. However, the cytoskeletal organization is not discernible at this or at high magnification ^19,20^. Scale bar: 500 nm. (D) Tubulin staining (green) of nephrocytes in *DotGal4>TubulinGFP* and CantonS (inset showing the partial punctate appearance of microtubules in nephrocytes). (E) Tubulin (green) staining in control (*DotGal4*) and *DotGal4>UAS Tubulin RNAi* nephrocytes. Graphic quantification of the decrease in the Tubulin intensity in the Tubulin knockdown nephrocytes. (F) Co-localization (white arrow) of Tubulin GFP (green) and Tubulin (red) in nephrocytes. Inset showing the co-localization index. (G) Tubulin staining in DMSO, Nocodazole treated and Nocodazole washout conditions in nephrocytes. Graph representing the quantification of Tubulin intensity after the Nocodazole washout compared to the control (DMSO) and Nocodazole treated nephrocytes. (H) ActinGFP (green) and Tubulin (red) partial co-localization (white arrow) at the cortex but not at the actin cluster (Inset: colocalization plots). Data representative of n ≥ 30 from three independent experiments. Scale bars: 10 µm. ER: endoplasmic reticulum, C: C bodies, FP: foot processes, MT: mitochondria.

**Fig. S2:**
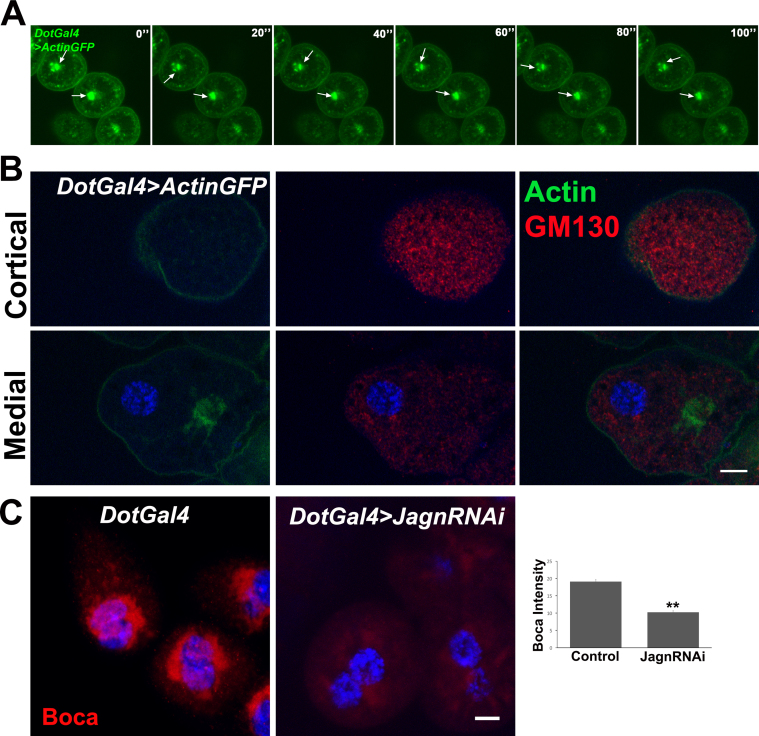
Actin cluster is stable and does not co-localize with Golgi apparatus. (A) Time series of still images extracted from live imaging of *DotGal4>UAS ActGFP* nephrocytes for 100 s indicating that medial actin clusters (white arrows) are stable over this time. (B) Actin (green) and GM130 staining (Golgi; red) in a nephrocyte showing no co-localization. (C) Boca (ER; red) staining in Control (*DotGal4*) and *DotGal4>UAS Jagunal RNAi* nephrocytes. Graph showing the quantification of the decrease in the Boca intensity in the JagnRNAi mutant. Scale bar: 10 µm.

Since podocyte foot process integrity depends on the maintenance of the actin architecture, we first examined the status of actin in *Drosophila* nephrocytes by staining for F-actin with Phalloidin in a wild type strain (Canton S) as well as using an ActinGFP reporter expressed in nephrocytes (*DotGal4>UAS Actin GFP*). Phalloidin staining in Canton S showed cortical actin at the cell periphery (Fig. 1A, arrowhead). In addition, all nephrocytes showed a medial cluster of densely packed actin located centrally in the cell, adjacent to nuclei (Fig. 1A, white arrow). In addition, GFP expression in *DotGal4>UAS Actin GFP* showed that it mirrored endogenous actin staining (Fig. 1B). This was also confirmed by double staining for Actin GFP and Phalloidin in *DotGal4>UAS ActinGFP* nephrocytes (Fig. S1A). A previous report had shown Phalloidin staining in nephrocytes, which also revealed a cytoplasmic localization although the details were not further investigated [21]. Live imaging of the actin cluster showed little dynamic activity, suggesting that this is a stable structure (Fig. S2A). To check whether the actin cluster is tethered to the cell membrane which lines extensive invaginations, we analyzed transgenic nephrocytes expressing ActinGFP along with membrane localized DsRed (mCD8-DsRed) (*DotGal4>UAS mCD8 Dsred>UAS ActinGFP*). An earlier report suggested that as the nephrocyte plasma membrane invaginates and forms lacunae from which there is rapid endocytosis, mCD8-DsRed is not restricted to the cell periphery but is also present in the cytoplasm [29]. However, the actin cluster showed no co-localization with the mCD8-DsRed, suggesting that this central actin is not tethered to the membrane (Fig. S1B). Thus we show for the first time that F actin is present in two compartments of the cell. Cortical actin may be equivalent to the foot process-like structure and cytoplasmic actin forms a cluster. Ultrastructural analysis by electron microscopy from our study (Fig. S1C) as well as other reports [21], [22] did not reveal the cytoskeleton organization of F-actin, rather the majority of the cellular area is occupied with different kinds of vacuoles possibly due to high rate of endocytosis.

### 2.2 Microtubules regulate actin cluster organization

Actin and MTs co-ordinate to maintain and organize cytoplasmic components and organelles [30], [31], [32], [33]. In podocytes, MTs are present in and maintain the primary foot processes [34], [7]. In nephrocytes, both GFP (*DotGal4>UAS Tubulin G*FP) and immunolabelling with beta-Tubulin shows that MTs usually appear punctate but in some regions appear filamentous (Fig. S1D, S1E). This was confirmed by double immunostaining for Tubulin GFP and beta-Tubulin (Fig. S1F). In the majority of insect and mammalian systems, MTs exist as filaments. Our analysis indicates that nephrocyte MT organization is not entirely filamentous and hence appears atypical.

To examine the organization of tubulin in nephrocytes as well as the role of MTs in the organization of actin, we assessed the effect of disrupting MTs. Nocodazole treated nephrocytes almost completely lacked tubulin staining and this effect is reversed by the washout of Nocodazole (Fig. S1G) suggesting that even punctate staining represents tubulin organized as MTs. Nocodazole treatment also caused disintegration of the medial actin cluster and decreased overall cortical actin (Fig. 1C, D), suggesting that the maintenance of actin requires intact MTs.

Co-staining of MTs and actin in control (*DotGal4>ActinGFP*) cells showed that although there was about 20% co-localization of the two cytoskeletal components at the cortex, the actin cluster was devoid of microtubules (< 2% co-localization) (Fig. S1H). This indicates that MTs regulate actin cluster organization in nephrocytes, but through an indirect mechanism.

### 2.3 The actin cluster is in close proximity to and is regulated by the endoplasmic reticulum

Cortical actin possibly represents the nephrocyte foot processes and it is directly linked to filtration function. In order to understand the significance of the medial actin cluster, we analyzed its localization pattern with respect to that of cellular organelles such as the endoplasmic reticulum and the Golgi apparatus. The actin cluster did not co-localize with the Golgi apparatus, identified by staining for GM130 (Fig. S2B), but was always found in close proximity to the ER, identified by the ER lumen marker Boca (Fig. 1E, F). Boca antibody [35] was validated by examining the expression status of Boca in an ER disrupted mutant such as Jagunal RNAi line (*DotGal4> UAS Jagunal RNAi*) (Fig. S2C)

To examine whether the ER plays a role in actin cluster organization, we perturbed ER organization by expressing *jagunal* RNAi [36] (Fig. S2C), and assayed for the effect on actin. Jagunal depletion (*DotGal4> UAS Jagunal RNAi*) severely disrupted the actin cluster as compared to control (*DotGal4*) whether assessed by staining for endogenous actin (Fig. 1G) or by overexpressed ActinGFP (Fig. 1H). This indicates that the ER also has an indirect role in regulating actin cluster organization.

### 2.4 A balance of Rac1 and Cdc42 activity is required for actin organization

**Fig. 2:**
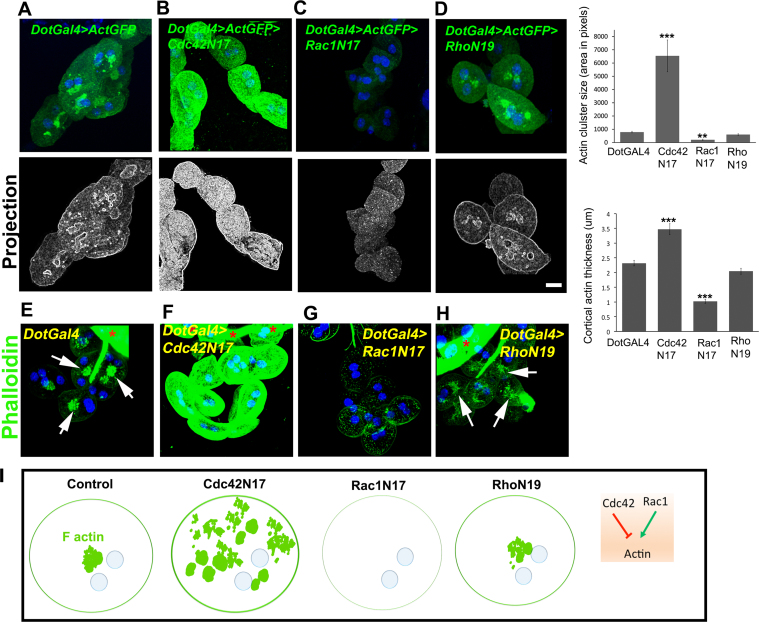
Rac1 and Cdc42 regulate actin cluster organization. (A-D) ActinGFP (green) in control (*DotGal4>ActGFP*) (A) and Cdc42N17 (B), Rac1N17 (C) and RhoN19 DN (D) nephrocytes as indicated. Graphs show that actin cluster size and cortical actin thickness were increased in Cdc42N17 and decreased in Rac1N17 cells. (E-F) Phalloidin staining for endogenous actin in control (*DotGal4*) (E) and Cdc42N17 (F), Rac1N17 (G) and RhoN19 (H) DN nephrocytes as indicated. White arrows indicate the presence of actin clusters and red asterisks indicate the proventriculus. (I) Models representing actin organization in control, Cdc42, Rac1 and Rho- DN expressing nephrocytes. Cdc42 DN causes increased cortical actin thickness and actin cluster size (green), whereas the Rac1 DN cells have thinner cortical actin and almost no actin cluster. Rho DN shows no change. n ≥ 30 cells from three independent experiments. Error bars depict ± SEM. **p < 0.01, ***p < 0.001. Scale bars: 10 µm.

We next asked how known direct regulators of actin might affect actin cluster positioning. RhoGTPases express ubiquitously in eukaryotes and are known to regulate intracellular actin organization and dynamics directly, and also affect cellular organelles and structure. To test the role of Rho-GTPases in nephrocytes, we impeded their activity by expressing dominant negative (DN) forms of Rac1 (*DotGal4>UAS-Rac1N17*), Cdc42 (*DotGal4>UAS-Cdc42N*17) and Rho A (*DotGal4>UAS-RhoN19*) and assayed for the effect on cortical actin and medial actin clusters (Fig. 2A–H). Quantitative analysis was done by measuring the size of actin cluster and thickness of cortical actin (see methods). Cdc42 DN nephrocytes showed several dispersed clusters with a significant increase in actinGFP intensity and increased size of each actin cluster (Fig. 2B), whereas lack of Rac1 activity obliterated the actin cluster (Fig. 2C). DN RhoA did not affect actin cluster organization (Fig. 2D). This was confirmed by Phalloidin staining of endogenous actin in nephrocytes expressing each dominant negative construct but not expressing ActinGFP (Fig. 2E–H). Thus Rac1 is essential for the formation and/or maintenance of the actin cluster and possibly for actin stability. Cdc42 has a negative effect on actin stability and could limit the size and distribution of the actin cluster. Upon Rac1 or Cdc42 inactivation, we also found that cortical actin thickness is noticeably affected, with decreased thickness in Rac1 DN and an increase in the Cdc42 DN nephrocytes. Hence, in nephrocytes, a balance of activity of Rac1 and Cdc42 is required for the unique actin organization (Fig. 2I). Our study shows that in *Drosophila* nephrocytes Rho-GTPases regulate actin organization by regulating different forms of actin filament formation. This demonstration of direct control of cytoskeletal organization by specific Rho GTPases in *Drosophila* nephrocytes lays the foundation for identifying specific regulators.

### 2.5 Nephrocyte diaphragm proteins also reside in the cytoplasm and regulate actin organization

**Fig. 3:**
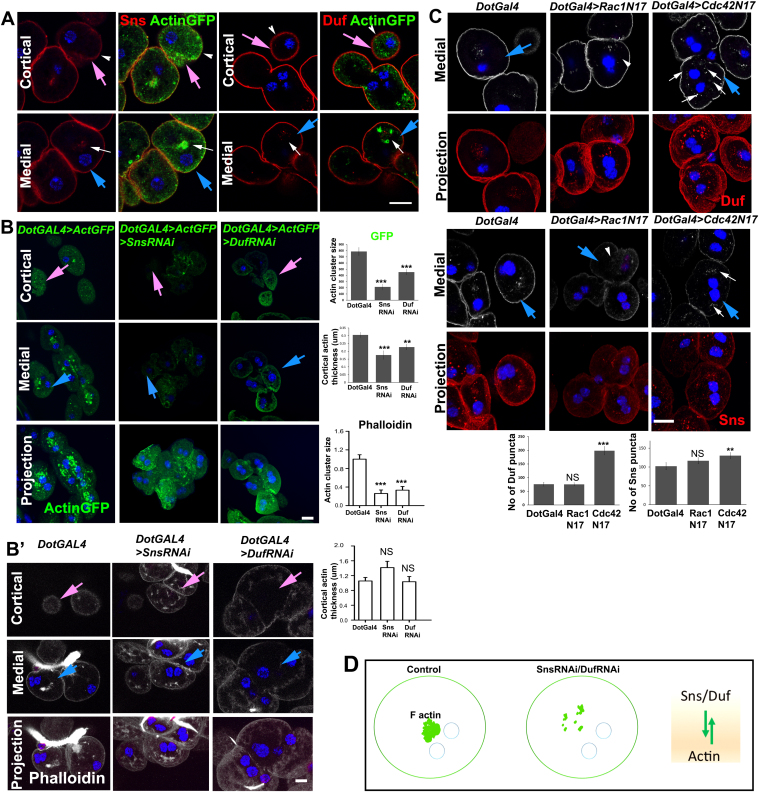
Actin organization and nephrocyte diaphragm protein localization are interdependent. A representative cell at the cortical (pink arrow) or medial (blue arrow) Z slice or projection is indicated. (A) ActinGFP (green) and Sns or Duf (red) co-staining in nephrocytes. ND proteins (red) and actin associate at the cortex (arrowheads) as well as at the cluster (arrows). (B) ActinGFP (green) organization in control (*DotGAL4>ActGFP*), Sns RNAi (*DotGAL4>ActGFP>SnsRNAi*) and Duf RNAi (*DotGAL4>ActGFP>DufRNAi*) nephrocytes. Graph represents quantification of actin cluster size or thickness in control and ND-RNAi nephrocytes. (B′) Endogenous actin (Phalloidin) organization in control (*DotGAL4*), Sns RNAi (*DotGAL4>SnsRNAi*) and Duf RNAi (*DotGAL4>DufRNAi*) nephrocytes. Graph represents quantification of actin cluster size or thickness in control and ND-RNAi nephrocytes. (C) Duf and Sns localization pattern in control, Rac1N17 and Cdc42N17 expressing nephrocytes. Duf and Sns cytoplasmic puncta are increased (arrows) in the Cdc42N17 and dispersed (arrowhead) in Rac1N17 nephrocytes. Graphs represent quantification of Duf and Sns puncta. (D) Models showing that actin cluster is perturbed in ND mutants. n ≥ 30 cells from three independent experiments. Error bars depict ± SEM. **p < 0.01, ***p < 0.001, NS: not significant. Scale bars: 10 µm.

**Fig. S3:**
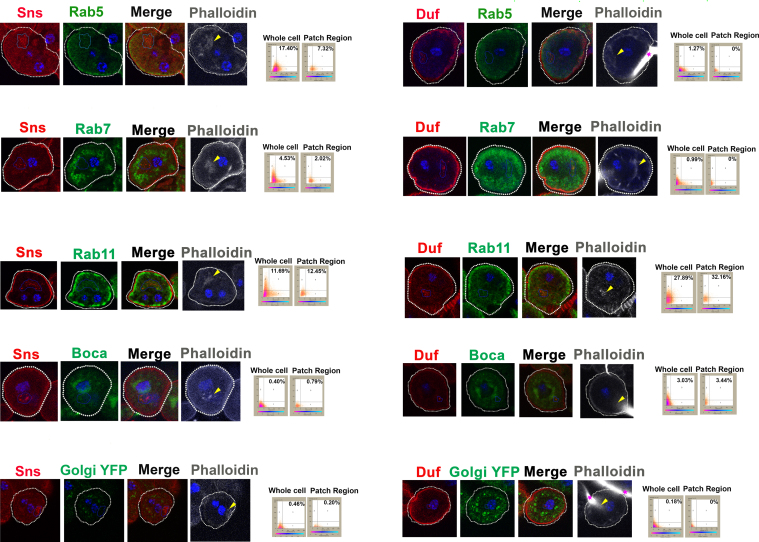
**Co-localization of Sns or Duf** with organelle markers detected by expression of Rab5GFP, early/sorting endosome (green in *DotGal4>UASRab5GFP)*, Rab7GFP, late endosome (green in *DotGal4>UASRab7GFP)*, Rab11GFP recycling vesicles (green in *DotGal4>UASRab11GFP)*, EYFP-Golgi (green in *DotGal4>UASGolgiEYFP)* or staining for the ER protein Boca (green in Canton(S)) as indicated. Cells were stained for either Sns (left panels) or Duf (right panels) (red) and counterstained with Phalloidin to detect endogenous actin. Nuclei labelled with DAPI. A single optical slice is shown. Graphs to the right of each set of panels indicate co-localization index for Sns or Duf (red) and the respective organelle marker (green) measured for the entire cell or for the cytoplasmic patch of Sns or Duf only. White dotted line indicates the cell boundary. Blue dotted line indicates Sns or Duf or actin patch as indicated. Phalloidin patch (yellow arrowhead) is shown for reference for each cell, but in some cases is an adjacent slice. Pink asterisk indicates background signal from extracellular material connecting the nephrocytes.

Actin and slit diaphragm proteins are critical for podocyte foot process architecture. Podocyte SD proteins, Nephrin and NEPH1 are transmembrane proteins that signal to activate a myriad of pathways to regulate actin organization in foot processes. Nephrocyte diaphragms are composed of proteins homologous to SD proteins, namely Kirre/ Dumbfounded (Duf) and Roughest (Rst) proteins (homologous to NEPH1); Hibris (Hbs) and Sticks and stones (Sns) (homologous to NPHS1) [22], [24]. However, their interaction with and ability to regulate the organization of actin have not been reported in nephrocytes. Hence, we tested the effect of perturbing representative ND proteins Sns and Duf, on actin organization. Sns and Duf are localized to cell membranes associated with the ND but reports also show a cytoplasmic component which has not been characterized [22], [21]. Through our protein localization analysis, we show that Sns and actin co-localize at the cell cortex and medial cluster whereas Duf co-localizes with cortical actin but lies in close proximity to the actin cluster (Fig. 3A). This indicates that there is a pool of intracellular diaphragm proteins, whose distribution overlaps with the actin cluster. Further, at the cell cortex, where foot processes and nephrocyte diaphragms are located, actin and ND proteins co-localize. Immunolocalization with vesicular (Rab5, Rab7, Rab11) and tubular (GolgiYFP, Boca for ER) organelle markers showed partial co-localization of SD proteins with Rab5 or Rab11, but not with any of the other markers (Fig. S3). This indicates that the intracellular pool of SD proteins is vesicular.

**Fig. S4:**
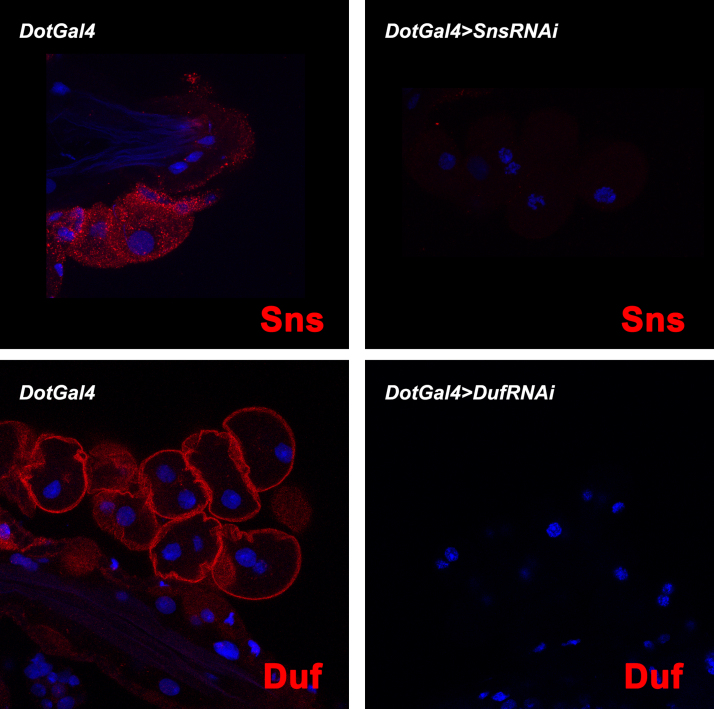
Validation of knockdown by *SnsRNAi* or DufRNAi determined by immunostaining (red) for Sns or Duf respectively, as compared to *DotGal4* controls. Nuclei marked by DAPI. Genotypes are as indicated.

Depletion of the ND proteins (*DotGal4>UAS SnsRNAi*; *DotGal4>UAS DufRNAi*) (Fig. S4) and detection of overexpressed ActinGFP (Fig. 3B) or endogenous actin without overexpression (Fig. 3B′) showed that lack of Sns or Duf caused a dramatic disorganization of medial actin. The cluster was disrupted and strongly reduced (Fig. 3B, B′). In contrast the cortical actin thickness was reduced only moderately when assessed by ActinGFP overexpression (Fig. 3B) and not significantly when endogenous actin was stained with Phalloidin (Fig. 3B′). Together these data indicate that aspects of actin organization are dependent on the ND proteins. We next tested whether the converse is true.

### 2.6 Rac1 and Cdc42 regulate ND proteins

Balanced activity of all the Rho-GTPases is essential for foot process organization as well as slit diaphragm arrangement in podocytes [37], [38]. Analysis of ND proteins Sns and Duf in DN Rho-GTPase- expressing nephrocytes showed that Rac1 inactivation led to dispersed Sns and Duf cytoplasmic punctae with no significant change in number. However, Cdc42 DN caused significantly more Sns and Duf puncta (Fig. 3C). This correlates well with the increased actin cluster phenotype seen upon Cdc42 inactivation. Thus, proper arrangement of the actin cluster is essential for the normal cytoplasmic localization of ND proteins. This shows that actin organization and ND protein localization are interdependent (Fig. 3D).

### 2.7 Sns and microtubules regulate each other in nephrocytes

**Fig. 4:**
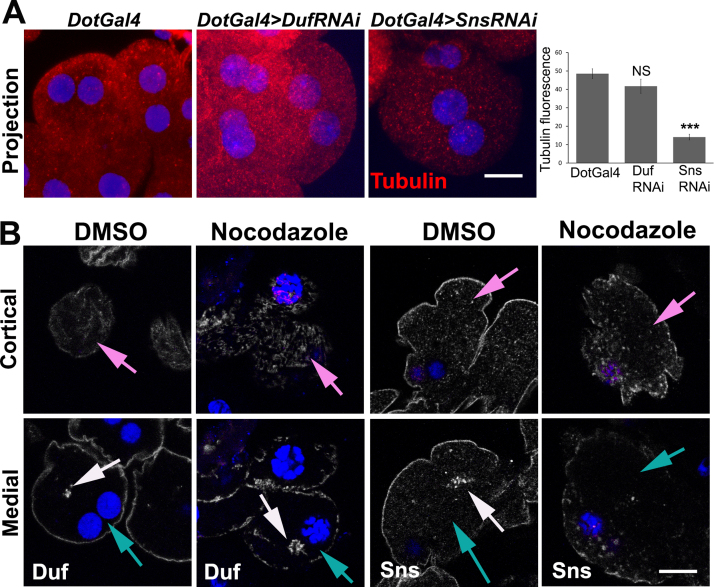
Sns and microtubules regulate each other in nephrocytes. A representative cell at the cortical (pink arrow) or medial (blue arrow) Z slice or projection is indicated. (A) Tubulin staining (red) in control, *Duf* RNAi (*DotGAL4>UASDufRNAi*) and *Sns* RNAi (*DotGAL4>UASSnsRNAi*) nephrocytes. Graph represents quantification of Tubulin fluorescent intensity. MTs are drastically reduced in the *Sns*RNAi but not in *Duf*RNAi nephrocytes. (B) Duf and Sns (white) staining in DMSO and Nocodazole- treated nephrocytes. Sns localization is disrupted in Nocodazole-treated cells while Duf distribution is only moderately affected (white arrows). n ≥ 30 cells from three independent experiments. Error bars depict ± SEM. ***p < 0.001, NS: not significant. Scale bars: 10 µm.

Our analysis shows that both tubulin and ND proteins regulate actin organization in nephrocytes. Hence we tested whether ND proteins and MTs affect each other's organization. Nephrocytes depleted of Sns but not of Duf, showed reduced beta- tubulin staining (Fig. 4A). Conversely, when MTs were disrupted using Nocodazole, there was a decrease in the cortical as well as the cytoplasmic Sns, while cytoplasmic Duf localization remained comparable to the control (Fig. 4B). This indicates that Sns and MTs are inter-dependent for their organization suggesting specificity in the interaction.

### 2.8 Actin cluster regulates endoplasmic reticulum morphology in nephrocytes

**Fig. 5:**
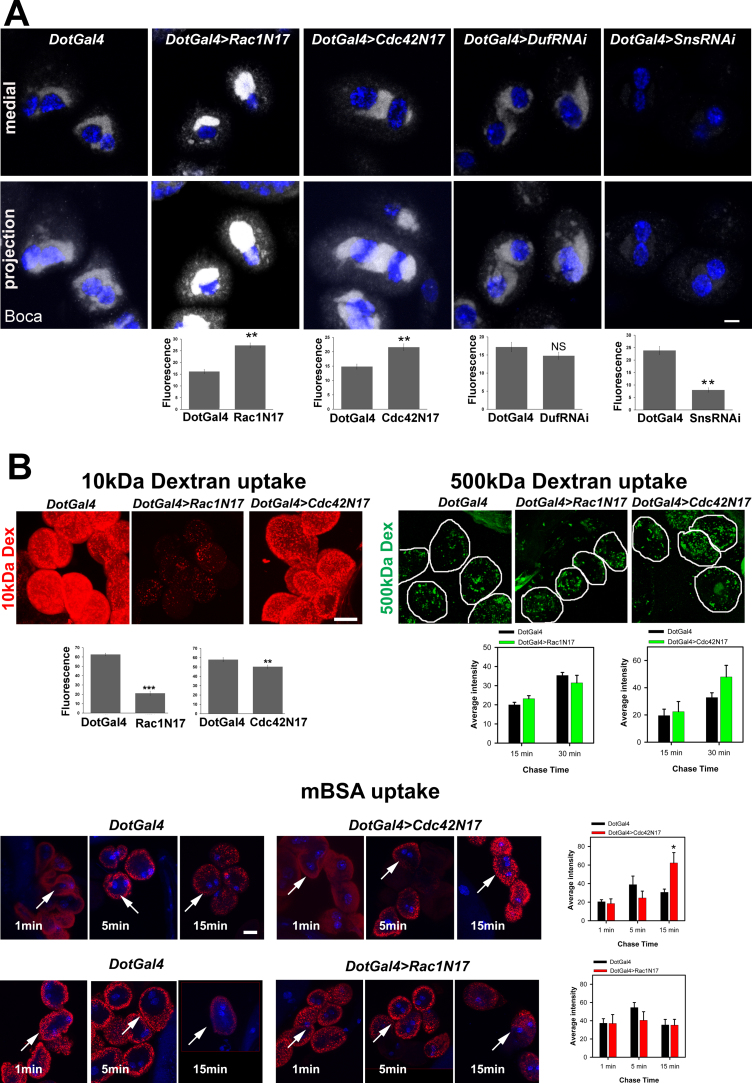
Actin cluster maintains endoplasmic reticulum organization and regulates ultrafiltration function in nephrocytes. (A) Boca (grey) staining in control and cells expressing DN constructs in a medial slice and projection. ER intensity was drastically increased in the Rac1 DN and Cdc42 DN mutant compared to the control. *Duf* RNAi did not affect the ER and *Sns*RNAi showed reduced ER. Graphs show the Boca fluorescence intensity in the control and mutants. (B) Uptake of 10 kDa Texas Red Dextran (red) or 500 kDa FITC Dextran (green) or mBSA (red) as indicated in control (*DotGal4*), Rac1N17 and Cdc42N17 expressing nephrocytes. 10 kDa Dextran uptake was reduced drastically in Rac1N17 and slightly in Cdc42N17 cells. 500 kDa Dextran uptake was not significantly changed even after prolonged incubation. mBSA uptake was more in Cdc42N17 nephrocytes as compared to the Gal4 control, but comparable to control in Rac1N17 nephrocytes. Representative images are shown. Arrows indicate examples of cells in the medial plane that were considered for quantitation. Graphs represent the quantification of total fluorescence intensity. n ≥ 30 cells from three independent experiments. Error bars depict ± SEM. *p < 0.05 **p < 0.01, ***p < 0.001, NS: not significant. Scale bars: 10 µm.

We found that actin cluster maintenance depends on the ER, but whether they are mutually dependent for nephrocyte organization is not known. The actin and MT cytoskeleton is known to play a role in maintaining the balance between ER tubules and sheets in many cell types [32]. To investigate whether the ER depends on actin organization and co-relate it with the presence of the actin cluster in nephrocytes, we stained for Boca in Rac1 DN, Cdc42 DN, or *Sns* and *Duf* RNAi mutants (*DotGal4>UAS-Rac1N17; DotGal4>UAS SnsRNAi*; *DotGal4>UAS DufRNAi; DotGal4>UAS-Cdc42N*17*; DotGal4>UAS-RhoN19*). The amount of ER was dramatically increased in the Rac1 DN and Cdc42 DN as estimated by quantifying staining intensity (see methods and Fig. 5A). As the Rac1 DN obliterated the actin cluster whereas Cdc42 DN resulted in increased actin (Fig. 2), this suggests that optimal levels of actin or cluster organization are essential for maintaining the ER.

Interestingly, *Duf* RNAi did not show a significant change but *Sns* RNAi showed drastic reduction of the ER (Fig. 5A). This correlates well with the extent of actin disruption in these mutants- reduction of Duf has a mild effect on the actin cluster, while Sns depletion greatly reduces cluster size and cortical actin thickness. These results suggest that ND protein-mediated actin organization is important in the maintenance of ER morphology in nephrocytes.

### 2.9 Rho-GTPases aid ultrafiltration

Disruption of MTs, ER, ND proteins or RhoGTPases (Rac1 or Cdc42) in nephrocytes, all result in perturbed actin organization. Since actin directly affects cellular architecture and the distribution of nephrocyte diaphragm proteins, we tested the effect of disrupting actin organization on nephrocyte function by assaying for ultrafiltration in dominant negative Rac1 or Cdc42 nephrocytes. Uptake of larger (500 kDa) Dextran molecules was not affected but uptake of 10 kDa Dextran was reduced (Fig. 5B). A time course (0', 15', 30') analysis with 500 kDa dextran also showed no significant difference in the uptake in dominant negative Rac1 or Cdc42 nephrocytes. However, using a tracer for receptor-mediated endocytosis, such as maleylated BSA (mBSA) we found that there was increased uptake in dominant negative Cdc42 nephrocytes, but not in dominant negative Rac1 nephrocytes. Thus Rac1 and Cdc42 differentially regulate size-based and charge-based ultrafiltration in nephrocytes indicating that organization of actin as well as ND proteins is important for determining filtration function.

## 3 Discussion

**Fig. 6:**
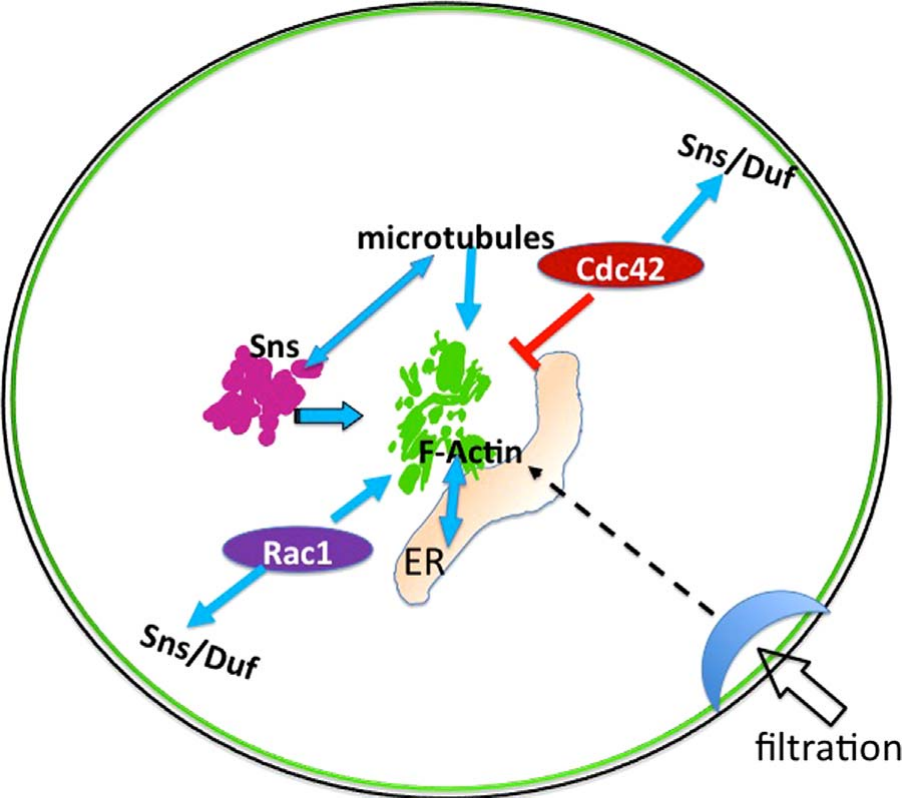
Model depicting nephrocyte ultrastructure and its regulation for ultrafiltration function.

In podocytes, perturbed RhoGTPase activity (and hence actin organization) leads to massive proteinuria and foot process effacement [39]. Our study elucidates the interlinked roles of intracellular organelles and nephrocyte diaphragm proteins in the regulation of the nephrocyte cytoskeleton for maintenance of its unique structure as depicted in Fig. 6. We provide previously undocumented evidence for the distribution of nephrocyte actin at the cell cortex and in the form of a central cluster. Since the nephrocyte membrane has extensive invaginations, forming lacunae, it is possible that the actin cluster is located close to the innermost lacunae. However, marking the membrane with mCD8 or transmembrane ND proteins such as Sns and Duf showed that the cluster is not associated with the in-foldings, but located deeper in the cytoplasm. Thus actin has a unique organization in the nephrocyte and the tight regulation of this cytoskeletal structure is required for nephrocyte filtration function. The more complex mouse podocyte architecture with secondary and tertiary foot processes is especially vulnerable to cytoskeletal defects and is dependent on Rac1, Cdc42 and RhoA. In contrast, we show that nephrocyte actin is maintained by the balanced activity of Rac1 and Cdc42, but not RhoA. In differentiated podocytes, Synaptopodin stabilizes Rho A by blocking proteasomal degradation to induce the formation of stress fibers in vitro [40], [18]. *Drosophila* has Tropomyosin, a functional ortholog of *synaptopodin* that is widely expressed. However, whether it stabilizes RhoA in nephrocytes is not known [50], [51]. If RhoA is rapidly degraded in nephrocytes, this GTPase might not play a role in actin organization in these cells.

Apart from small GTPases, the large GTPase Dynamin is also expressed in podocytes where it resides in the vesicles present in the foot processes [41]. Many reports indicate the significance of Dynamin in actin filament formation through its interaction with actin binding proteins such as Profilin, Nck and the capping protein Gelsolin [42]. Mice deficient for podocyte *dynamin 1* and *dynamin 3* have proteinuria and foot process effacement [43], [44]. In *Drosophila* nephrocytes, *shibire* (Dynamin) is required for micropinocytosis [27] and *shibire* mutants show elongated labyrinthine channels [45]. The specific regulation of the nephrocyte cytoskeleton by Dynamin would be an interesting aspect to explore.

In nephrocytes Sns and Duf co-express and are essential for diaphragm and foot process formation [22]. However, in *Drosophila* eye and muscle cells, Sns and Duf are expressed in two different cell types and function in a complementary fashion [46], [47], [48]. Our study additionally documents the significance of cytoplasmic Sns and Duf in regulating actin organization, which could be direct or a result of altered cortical actin when ND proteins are lost or reduced. Further, our data indicate that Sns has a more significant role than Duf in nephrocyte architecture and function. Though the level of knockdown achieved for both Sns and Duf is comparable, the severity of the phenotype differs. It should be noted that Sns and actin co-localize at the medial cluster whereas Duf lies in close proximity to the actin cluster (Fig. 3A). Hence it is likely that the roles of the two proteins in organizing actin may be different, explaining the stronger effects on Sns perturbation than on Duf perturbation.

Sns and Duf are transmembrane proteins, and hence not expected to reside in the cytoplasm. However, it is possible that the cytoplasmic clusters contain truncated versions of the proteins or full-length proteins present on vesicles. Further analysis such as immuno electron microscopy will be required to establish details of their distribution. Cultured kidney podocytes also show punctate, cytoplasmic localization of SD proteins, which partially overlap with the actin stress fibers [49]. Thus cytoplasmic co-localization is conserved but the significance of this in podocytes is not known yet.

The intricate connection between the ER and cytoskeleton has been established in several cell types. Actin and MTs associate with the ER in a specialized manner forming a network to regulate ER structure [32]. In this study, we report the relationship between the ER and nephrocyte cytoskeleton. The actin cluster lies adjacent to the ER and is required for proper ER organization. If the cluster is mislocalized the ER expands. Similarly, Sns plays a major role in ER maintenance possibly through direct or indirect regulation of MTs and actin. Thus multiple structural components interact to maintain the complex and specialized architecture of the nephrocyte, thereby regulating efficient filtration function. Interestingly, perturbation of actin with different RhoGTPases had distinct effects on ultrafiltration function. While Cdc42N17 expressing nephrocytes had reduced uptake of 10 kDa Dextran and increased uptake of mBSA, Rac1N17 expressing nephrocytes had reduced uptake of 10 kDa Dextran and relatively normal mBSA uptake. Cdc42N17 expression caused increase in cortical actin which could impede fluid phase uptake, but may not hinder receptor mediated endocytosis. It is possible that entrapment of the probe cargo in the cortical actin could lead to accumulation of the signal over time, leading to increased fluorescence intensity. On the other hand Rac1N17 causes reduced cortical actin, which does not seem to hinder fluid phase uptake of large cargo, but affects smaller cargo. Thus the difference in ultrafiltration phenotypes upon expression of dominant negative RhoGTPases is likely due to dramatic effects on the nephrocyte architecture.

In summary, our findings elucidate the conserved molecular interactions in nephrocytes that are essential for ultrafiltration function. This study is relevant to kidney podocytes where slit diaphragm proteins and the cytoskeleton play significant roles in filtration function, but the crosstalk between the cytoskeleton and ER is largely unexplored. Our analysis provides fundamental information required to probe further the crosstalk between cytoskeletal elements, the ER and ND proteins, which could aid analysis of podocyte biology and kidney disease.

## 4 Methods

### 4.1 *Drosophila* stocks used

Canton- S, *UAS Actin GFP (III)* (Bloomington 9257); *UAS TubulinGFP* (Bloomington 7373); UAS *Rac1N17* (Bloomington 6292); *UAS Cdc42N17* (Bloomington 6288); *UAS RhoAN19* (Bloomington 7328); *UAS snsRNAi*; *UAS dufRNAi*, *UAS jagunalRNAi* (VDRC 108991), *UAS tubulin RNAi* (VDRC 33427), *UAS DotGal4* (Bloomington 6903), *UAS Rab5GFP*, *UAS Rab7GFP*, *UAS Rab11GFP* (kind gift from Prof. Marcos Gonzalez Gaitan, University of Geneva), P{sqh-EYFP-Golgi}3 (Bloomington 7193).

### 4.2 Immunostaining and immunofluorescence analysis

Dissected nephrocytes were immunostained with desired primary and secondary antibodies as described before (Das et al., 2008c). Phalloidin staining (660 nM) was performed post 0.3% Triton-X permeabilization. Antibodies used: Chick- and rabbit- anti-GFP (Invitrogen, 1:500); mouse anti-Tubulin (E7s, DSHB, 1:100); Rat anti-Duf (Eyal Schejter, Weizmann Institute of Science, Israel; 1:200); Rabbit anti-Sns (Susan Abmayr, SIMR, USA; 1:100), Guinea pig anti Boca (1:100) (Richard Mann, Columbia University, USA). Phalloidin-Alexa568 or Phalloidin-Alexa633 (Invitrogen, 1:100) was used to detect Actin. Imaging was on Zeiss LSM-Meta510 or LSM780 or LSM880. Equal numbers of sections were projected for each image shown.

### 4.3 Nocodazole treatment assay

Dissected nephrocytes (N = 10 larvae) were incubated in S2 medium containing various concentrations of Nocodazole or DMSO (Sigma Aldrich) for 6 h at room temperature, fixed in 4% paraformaldehyde and immunostained. 1 mM Nocodazole was selected for experiments based on anti-beta Tubulin staining. The cell viability was checked by Nocodazole washout experiment where Nocodazole was removed after 6 h and the cells were kept in Schneider's S2 medium for 2 h at room temperature to allow the reformation of microtubules. This was followed by immunostaining protocol for Tubulin detection (Fig S1G).

### 4.4 Quantification and statistical analysis

n ≥ 30 cells for each genotype in each experiment. Actin cluster quantitation was done by calculating the particle size in terms of area using Image J software. Cortical actin thickness was measured using LSM Image analyzer by calculating the length in microns from the plasma membrane to the internal limit of cortical actin in the medial slice. Average values were plotted using Microsoft Excel. Statistical significance was calculated using single factor ANOVA (analyses of variance) with Microsoft Excel (P < 0.01 was considered significant).

### 4.5 Ultrafiltration assay

Refer to Weavers et al. [22] for 10 kDa dextran. For 500 kDa dextran uptake, cells were incubated in FITC-Dextran for 0, 15 and 30 min, washed, fixed and then imaged. Cy3mBSA uptake was done as in [27]. Cells were incubated for 1 min in 0.33 mg/ml mBSA_Alexa555, washed and then fixed after 1, 5 or 15 min at room temperature, followed by imaging. Each experiment was repeated three times. A total of at least 50 cells from 8 larvae were analyzed per genotype. For quantification of uptake ten optical slices (total thickness: 3 µm) from the medial plane were considered for each cell. Only those cells and images where the medial plane was seen were quantified.

### 4.6 Co-localization index calculation

Single optical slices were analyzed using the Histo option in Zeiss LSM examiner software and the co-localization graphs were plotted.

For co-localization of Sns or Duf with organelle markers, P{sqh-EYFP-Golgi}3 was used for marking Golgi or *DotGal4* was used to drive expression of respective organelle markers (*UAS Rab5GFP*, *UAS Rab7GFP*, *UAS Rab11GFP)* in nephrocytes and stained with anti-GFP. For detection of ER, wild type nephrocytes were stained with Boca. The co-localization index was calculated separately for the total cell area and for the cytoplasmic Sns or Duf cluster. Where required, images for representation were adjusted equally for brightness/contrast using Adobe Photoshop Elements14.
